# Mechanistic Insights into the Aging and Regeneration of SBS-Modified Asphalt Under Coastal Humid–Hot Environmental Conditions

**DOI:** 10.3390/ma19153221

**Published:** 2026-07-28

**Authors:** Chien-Ta Chen, Ayad Thabet Saeed Alghabsha, Xinxin Cao, Jiaolong Ren

**Affiliations:** 1 School of Intelligent Construction, Fuzhou University of International Studies and Trade, Fuzhou 350202, China; chenjianda@fzfu.edu.cn (C.-T.C.); ayade@fzfu.edu.cn (A.T.S.A.); 2 College of Metropolitan Transportation, Beijing University of Technology, Beijing 100124, China; caoxinxin@sdut.edu.cn; 3 School of Civil Engineering and Geomatics, Shandong University of Technology, Zibo 255000, China

**Keywords:** SBS-modified asphalt, coupled aging, regeneration, industrial animal oil, waste engine oil, coastal humid–hot environmental condition

## Abstract

The deterioration of SBS-modified asphalt under coupled temperature–ultraviolet (UV)–coastal humidity conditions is significantly accelerated in coastal regions because of seawater evaporation, leading to severe durability degradation of pavement materials. However, the performance evaluation laws and underlying regeneration mechanisms under such coupled environmental aging conditions remain insufficiently understood. Therefore, taking Shanghai as a representative coastal city, a temperature–UV–coastal humidity coupled aging system was established to simulate the saline and humid environment of coastal regions. Industrial animal oil and waste engine oil were selected as regeneration materials, and a multi-scale experimental approach was adopted to evaluate the performance recovery of aged asphalt. The results indicate that both regeneration materials effectively restore ductility and improve rheological behavior, while reducing viscosity but cause a measurable decrease in softening point, indicating a reduction in high-temperature stability. Industrial animal oil shows superior improvement in ductility, whereas waste engine oil exhibits stronger effects on viscosity reduction and microstructural regulation. A content of approximately 6% was recommended as a practical content to balance performance recovery and high-temperature stability under the tested coupled-aging condition. Microstructural analysis confirms that the regeneration mechanism is dominated by light component replenishment and colloidal structure reconstruction rather than chemical modification of SBS chains.

## 1. Introduction

Asphalt pavements in coastal regions are not only exposed to temperature and ultraviolet (UV) but are also continuously influenced by coastal humidity resulting from seawater evaporation [1]. Under the additional effect of a high-salt humid environment, asphalt binders undergo a distinct aging process that differs from conventional inland environmental aging [2], resulting in accelerated deterioration of mechanical and rheological performance [3,4]. SBS-modified asphalt is one of the most widely used high-performance binders in high-grade asphalt pavements because of its excellent high- and low-temperature properties [5,6,7]. Therefore, clarifying the aging evolution, regeneration efficiency, and performance recovery mechanism of SBS-modified asphalt under coastal temperature–UV–coastal humidity coupled conditions is essential for improving the durability evaluation and maintenance design of asphalt pavements in coastal regions [8,9]. In engineering practice, SBS-modified asphalt is commonly evaluated through conventional binder performance tests, including penetration, softening point, ductility, elastic recovery, viscosity, and storage stability. These indicators are generally used for material acceptance, construction quality control, and pavement maintenance evaluation. All relevant tests involved in this study were conducted in accordance with the Chinese Standard “Standard Test Methods of Asphalt and Asphalt Mixtures for Highway Engineering (JTG 3410-2025)” [10].

Previous studies have investigated the aging behavior of SBS-modified asphalt under different environmental actions. For example, Xiong et al. [11], Yu et al. [12], Liu et al. [13], and Zeng et al. [14] conducted a simulation of the ultraviolet aging process of modified asphalt to assess the UV aging behavior. Geng et al. [15] and Liu et al. [16] employed the dry-wet cycling aging method to simulate the aging process of SBS-modified asphalt under the influence of moisture. The results indicated that the effect of moisture would significantly impact the physical properties of SBS-modified asphalt. Khalighi et al. [17] analyzed the effects of liquid water and water vapor on the aging of asphalt binders, with the aim of developing more durable road surfaces. However, most existing research has focused on single-factor thermal aging conditions, while the synergistic effects of temperature, UV radiation, and moisture—especially under high salt and humidity conditions—remain insufficiently understood.

Due to the scarcity of high-quality asphalt resources in recent years, the regeneration of aged asphalt has become an important engineering issue. To restore the performance of aged asphalt, various regeneration technologies have been widely investigated in recent years, including the use of waste oils, bio-based oils, and industrial by-products. These regeneration materials have been proven to effectively replenish light components, improve low-temperature flexibility, and partially restore the rheological properties of aged asphalt [18,19]. Li et al. [20] investigated the effects of different proportions of waste cooking oil and waste engine oil on the regeneration performance of hot-aged asphalt. Chen et al. [21], Karamroudi et al. [22], Kumar et al. [23], and Bai et al. [24] investigated the regenerative effect of used engine oil on the technical properties of aged asphalt. However, most existing studies are still limited to thermally aged asphalt systems, and the regeneration behavior under coupled environmental aging conditions, particularly in coastal humid–hot climates, remains insufficiently understood.

In addition, microstructural mechanisms governing the aging and regeneration process are another important focus from the point of view of asphaltene de-aggregation, functional group evolution, colloidal structure regulation, and SBS network reconstruction [25,26]. For example, Xu et al. [27] proposed a regeneration mechanism involving control of the colloidal structure and structural reconfiguration of SBS polymers. They also verified the regeneration effect by combining fluorescence images, functional group characteristics, and molecular weight distribution. Shi et al. [28,29] investigated the effects of active regenerants on the rheological properties and microstructure of SBS-modified asphalt. However, most studies have focused on conventional thermally aged asphalt systems and their regeneration. The relationship between chemical structure restoration, micro-phase evolution, and macroscopic performance recovery of SBS-modified asphalt under coastal coupled aging conditions remains insufficiently understood.

Therefore, this study establishes a temperature–UV–humidity coupled aging system using simulated seawater to represent coastal saline–humid conditions. Industrial animal oil and waste engine oil are used as regeneration materials for aged SBS-modified asphalt, and a multi-scale approach combining conventional tests, FTIR, AFM, and fluorescence microscopy is adopted to evaluate performance recovery and microstructural evolution.

## 2. Materials and Method

### 2.1. Materials

(1) Asphalt

The SBS-modified asphalt material was provided by the Qilu Petrochemical Company (Zibo, China) in this study. The conventional physical properties of the SBS-modified asphalt used in the experiments were measured, and the main properties are shown in Table 1.

(2) Industrial animal oil

The industrial animal oil used was extracted from the fat tissues during the meat processing process through processes such as boiling and separation in this study. It was provided by the Guangzhou Fufei Chemical Technology Co., Ltd. (Guangzhou, China). The main performance indicators of industrial animal oil are presented in Table 2.

(3) Waste engine oil

The regeneration material used was waste engine oil in this study. It was obtained from the discarded oil that was replaced during the maintenance of small cars with a driving range of approximately 5000 km. After filtration and heating treatment, it was used in the regeneration experiment. The main performance indicators of waste engine oil are presented in Table 3.

### 2.2. Preparation Method of Asphalt

(1) Preparation of aged asphalt

**Figure 1:**
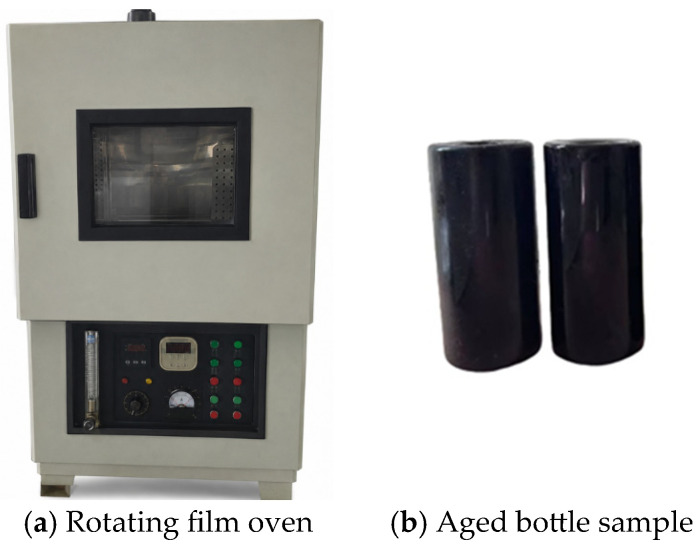
Rotating film oven and aged bottle sample.

A HW-85 type rotating film oven was used by Cangzhou Huarui Instrument & Equipment Co., Ltd. (Hejian, China) to conduct short-term aging treatment on the asphalt to simulate the early thermal-oxidative aging process that the asphalt underwent during mixing, transportation, and paving. The rotating film oven and the aged bottle samples are shown in Figure 1.

The specific steps were as follows:

- Firstly, 35.0 ± 0.5 g of the SBS-modified asphalt sample was weighed and poured into a clean aging bottle.
- Subsequently, the aging bottle containing the sample was installed on the fixed position of the circular support of the oven. A precise flowmeter was used to adjust the air flow to the specified requirement of 4000 ± 200 mL/min, and the temperature was controlled at 163 °C. Heating was maintained for 85 min.
- After the aging process was completed, the RTFOT was turned off, the aging bottle was quickly removed, and the sample was poured into a pre-prepared round disk container. To ensure the consistency of the samples for the subsequent long-term aging test, the asphalt mass in each round disk container was controlled at around 80 g.
- Finally, the round disk containing the aged asphalt was placed in a dust-free environment and allowed to cool to room temperature.

**Figure 2:**
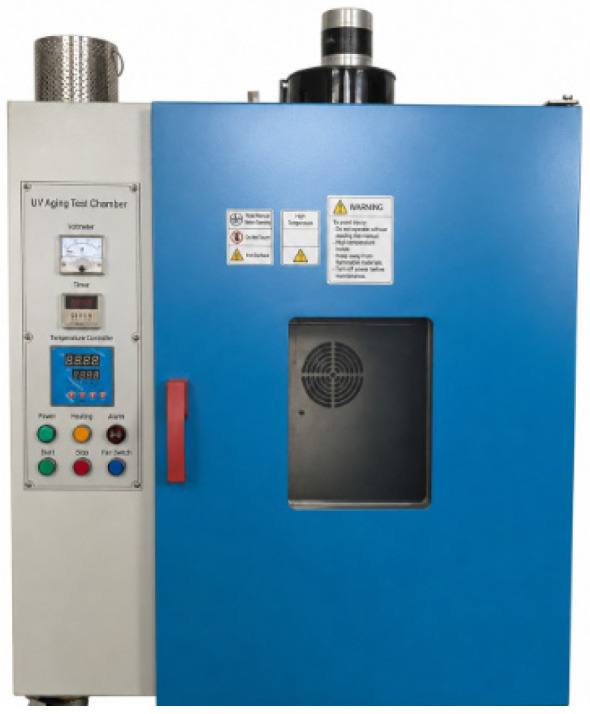
Multi-environment coupled aging chamber.

This study conducted a long-term aging test on asphalt samples that had undergone short-term aging using a multi-environment simulation aging chamber. The multi-environment coupling aging chamber is shown in Figure 2.

The specific steps were as follows:

- Firstly, the asphalt samples that had undergone short-term aging treatment were placed on the platform support in the aging chamber. The design capacity of this aging test was capable of accommodating up to 6 circular containers.
- Subsequently, the aging conditions of the aging chamber were set. This multi-environment coupled aging chamber had a temperature control range of 10 °C to 80 °C. Through an accurate temperature control system, the environmental temperature was stably adjusted to meet the thermal aging requirements of different aging stages.
- In addition, six 40 W UVA-340 ultraviolet lamps by Shenzhen Guanhongrui Technology Co., Ltd. (Shenzhen, China) with a wavelength range of 320–400 nm were installed on the top of the aging chamber. This wavelength band was consistent with the most destructive ultraviolet spectrum of sunlight on asphalt, accurately simulating the radiation intensity of ultraviolet rays in the operating environment.
- At the same time, the height of the platform support was approximately 15 cm from the lamp tubes, ensuring that the intensity of ultraviolet radiation on each sample was consistent. When simulating the erosion of asphalt surface by rainfall, it was sprayed regularly according to the set water volume and time intervals.
- Finally, after the aging test was completed, the circular disc container with asphalt was moved to a dust-free environment and allowed to naturally cool to room temperature, so that subsequent macroscopic performance tests and microscopic characterization could be carried out.

(2) Preparation of regeneration asphalt

The preparation process of regeneration asphalt was as follows:

- First, the asphalt samples that had undergone temperature-UV-humidity coupled aging were placed in a 160 °C constant temperature oven and heated until they completely melted.
- Subsequently, industrial-grade animal oil and waste engine oil were selected as the regeneration materials and were respectively added to the aged asphalt. The mixture was stirred with a glass rod for 1 min to achieve preliminary blending, and then a LR10 high-speed shear machine was used by Hebei Hangxin Instrument Manufacturing Co., Ltd. (Baoding, China) to continuously shear at 1500 r/min for 20 min to ensure the full integration of the regeneration materials with the aged asphalt.
- After the high-speed shearing process was completed, to eliminate the bubbles that might have been introduced during the shearing process and to ensure that the molecules of the regeneration materials had sufficient time to migrate, penetrate, and complete the sufficient physical and chemical reactions with the aged asphalt components, the regeneration asphalt samples were transferred to a room temperature environment and left to stand for more than 8 h. The final prepared regeneration asphalt samples were used for subsequent performance tests and analysis.

### 2.3. Method

#### 2.3.1. Softening Point

**Figure 3:**
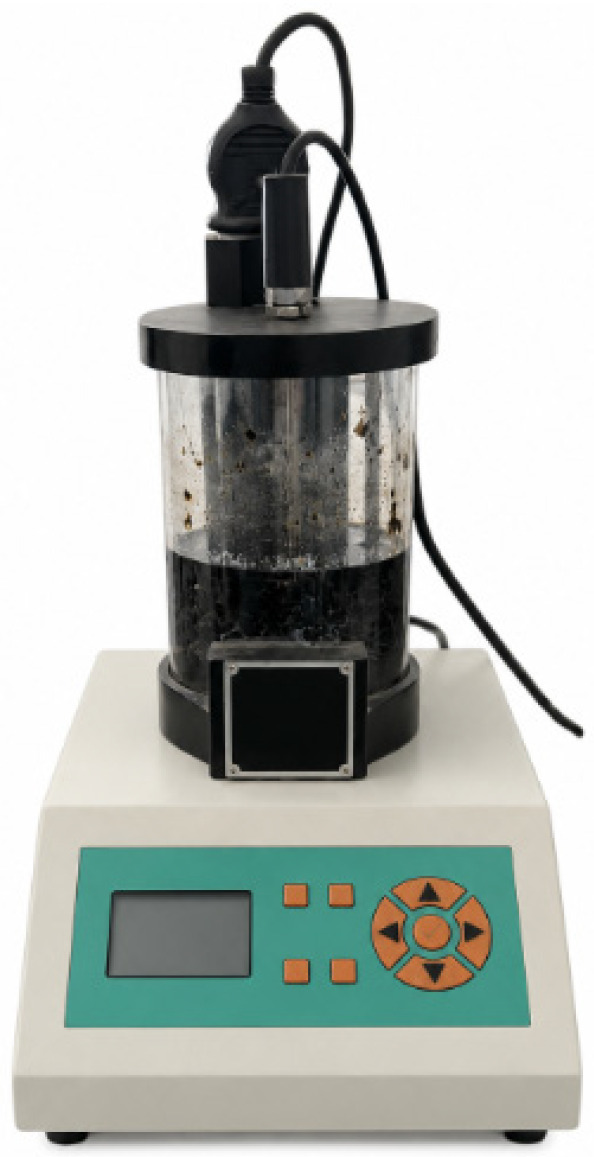
Softening point tester.

The softening point of the asphalt was tested using an SYD-2806E-type softening point instrument by Hebei Luchang Keyu Instrument Company (Cangzhou, China). The sample ring and sample plate containing asphalt were placed in a constant temperature water bath at 5 ± 0.5 °C for 15 min. Then, the asphalt was placed in the softening point tester, the steel ball was inserted, the equipment was turned on, and the test was conducted. The heating rate was set within the range of 5 ± 0.5 °C/min. At this time, the asphalt gradually began to soften and fall as it was heated. The temperature at which the steel ball precisely landed on the barrier was defined as the softening point. The softening point tester is shown in Figure 3.

The softening point increase ratio (*PI*) represents the percentage difference between the softening point after aging and the softening point before aging. *PI* was calculated according to Equation (1).

(1) $$PI=\frac{SP}{SP_{0}}\times 100\%$$

where *SP*_0_ represents the softening point before aging (°C); *SP* represents the softening point after aging (°C).

#### 2.3.2. Ductility

**Figure 4:**
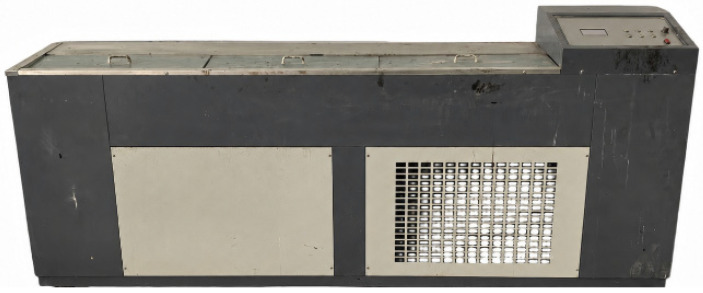
Elongation meter.

The low-temperature elongation property of asphalt was tested using an FYY-7 type elongation tester by Hebei Luchang Keyu Instrument Company (Cangzhou, China). The test mold containing the asphalt and the base plate were placed in a constant temperature water bath at 5 ± 0.5 °C for 90 min. Then, the stretching was carried out at a constant rate of 1 ± 0.5 cm/min in a 5 °C constant temperature water bath, and the average values of the three groups of stretching lengths at the time of sample fracture were recorded as the elongation index. The elongation meter is shown in Figure 4.

The ductility retention index (*DI*) represents the percentage of the elongation value after aging compared to the elongation value before aging. *DI* was calculated using Equation (2).

(2) $$DI=\frac{D}{D_{0}}\times 100\%$$

where *D*_0_ represents the ductility before aging (mm); *D* represents the ductility after aging (mm).

#### 2.3.3. Viscosity

**Figure 5:**
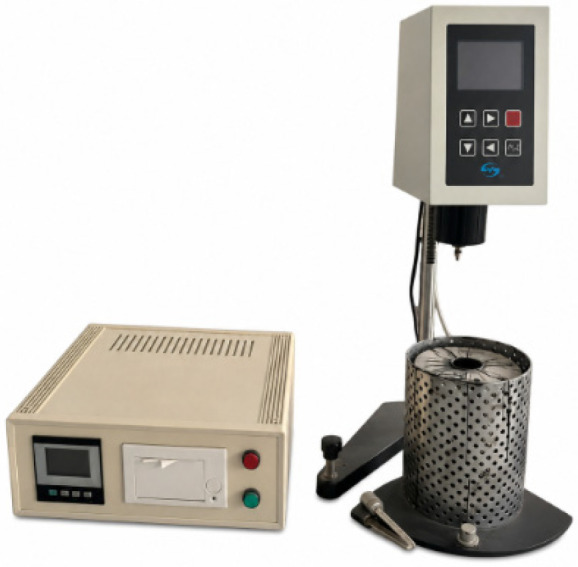
Viscometer.

The apparent viscosity of SBS-modified asphalt was measured by the rotational method of a Brookfield viscometer by Shanghai Changji Geological Instrument Co., Ltd. (Shanghai, China), and the flow performance of the asphalt was evaluated using an NDJ-1C type viscosity tester by Shanghai Changji Geological Instrument Co., Ltd. (Shanghai, China). In this study, the test temperatures of 115 °C, 135 °C, 155 °C, and 175 °C were selected. The appropriate rotor and rotational speed were determined based on the estimated viscosity. Before the test, the rotor and the sample cylinder were placed in the target temperature for 1.5 h of constant heating. After installation, the temperature was maintained at the target value for no less than 15 min. Then, the viscosity tester was started. After the rotational speed was stabilized and the reading was stable, data were recorded every 60 s three consecutive times. Finally, the average value was taken as the apparent viscosity result. The viscometer is shown in Figure 5.

The viscosity ratio (*VR*) represents the percentage difference between the viscosity value after aging and the viscosity value before aging. *VR* was calculated according to Equation (3).

(3) $$VR=\frac{\eta }{\eta_{0}}\times 100\%$$

where *ƞ*_0_ represents the viscosity before aging (Pa·s); *ƞ* represents the viscosity after aging (Pa·s).

#### 2.3.4. FTIR

**Figure 6:**
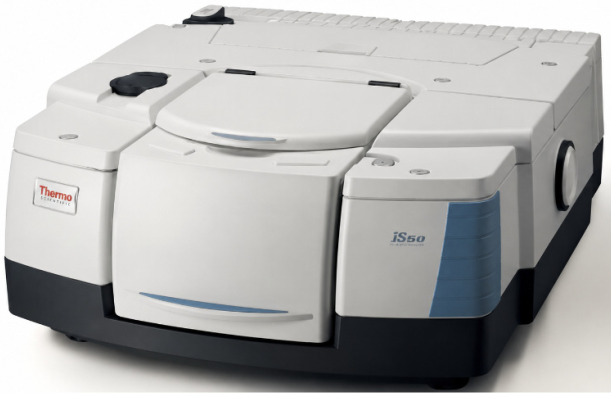
Fourier infrared spectrometer.

This study employed a Nicolet 5700 Fourier Transform Infrared Spectrometer (Thermo Electron Scientific Instruments Corporation, Madison, WI, USA) for the experiment. It enabled the acquisition of infrared spectrograms of asphalt under various aging conditions, and the absorption peaks within the spectrum were analyzed using it. The wave number range was from 400 to 4000 cm^−1^, with a resolution of 4 cm^−1^. The Fourier infrared spectrometer is shown in Figure 6.

#### 2.3.5. AFM

**Figure 7:**
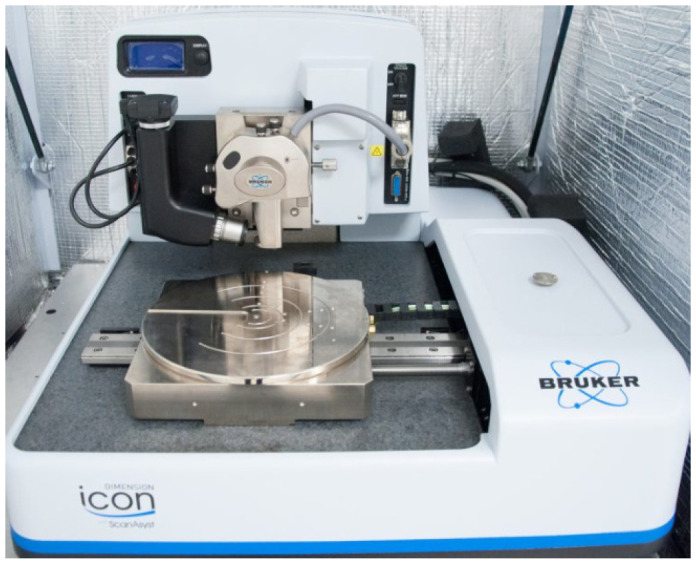
AFM instrument.

In this study, the tapping mode was selected. The AFM instrument used the Dimension Icon model produced by BRUKER (Santa Barbara, CA, USA), and an atomic force microscope with an RTESPA-150 probe (Bruker AFM Probes, Camarillo, CA, USA) was chosen. The elastic constant of the probe was 40 N/m, the scanning rate was 0.5 Hz, the scanning range was 20 μm × 20 μm, and the number of scanning points was 256 × 256. The AFM instrument is shown in Figure 7.

#### 2.3.6. FM

**Figure 8:**
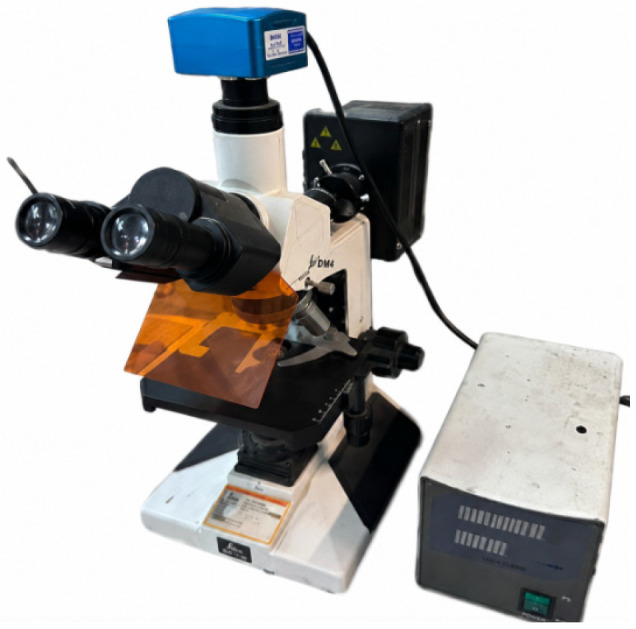
Fluorescence microscope.

This study employed an LW100 FT/B type fluorescence microscope by Shangguang Instrument Co., Ltd., (Suzhou, China), with the magnification set at 400 times. The sample was prepared by using the hot drop method. The fluorescence microscope instrument is shown in Figure 8.

The detailed steps were as follows:

- The SBS-modified asphalt was heated to 160 °C to achieve a molten and flowing state and was continuously stirred to ensure uniform heating of the sample.
- A glass rod was dipped into a small amount of asphalt and quickly dropped onto the center of a clean glass slide. The size of the asphalt drop was kept moderate; usually, 3–5 mm in diameter was appropriate.
- Before the asphalt cooled and solidified, it was quickly covered with a cover glass and appropriate pressure was applied to make the asphalt spread evenly and form a film.
- The sample was allowed to cool naturally to room temperature to fully solidify the asphalt. The generation of bubbles during cooling was minimized to ensure an observation effect.

### 2.4. Determination of Coupled Aging Conditions

#### 2.4.1. Temperature

According to the survey data from the National Environmental Information Center of China [30], July is usually the month with the highest average temperature in Shanghai, during which asphalt pavements are subjected to the most significant thermal aging effect. Based on statistical analysis of the average maximum summer temperature in Shanghai from 2001 to 2020 and considering that the surface temperature of asphalt pavement in summer is generally about 30–35 °C higher than the air temperature, the pavement temperature in Shanghai can reach approximately 60–70 °C under high-temperature summer conditions. Therefore, the thermal aging temperature in this study was set at 70 °C to simulate the high pavement temperature condition in coastal humid–hot regions. To ensure that all asphalt samples were subjected to the same duration of thermal action, the total thermal aging time was set as 145 h.

#### 2.4.2. UV

According to the survey data from the National Environmental Information Center of China [31], summer has the highest solar altitude angle and the longest sunshine duration, and asphalt pavement receives the strongest UV radiation during this period. The UV radiation in summer accounts for a relatively high proportion of the annual UV exposure. Considering the attenuation of solar radiation during atmospheric transmission, the actual UV radiation received by the pavement surface was estimated as a certain proportion of the total summer solar radiation.

In this study, the aging chamber was equipped with six 40 W UVA-340 ultraviolet lamps, with a wavelength range of 320–400 nm and a total radiation intensity of 240 W/m^2^. A UVA-340 lamp was selected because its wavelength range is close to the UV band that has a strong aging effect on asphalt materials. According to the energy equivalence principle, the indoor UV aging time was calculated using Equation (4), in which the indoor UV radiation intensity multiplied by the indoor exposure time is equivalent to the natural UV radiation energy received outdoors. Based on this conversion, the UV aging duration was set as 80 h.

(4) $$t=\frac{F\times b}{3600\times Q}$$

where *F* represents the average total solar radiation in summer (J/m^2^); *b* represents the proportion of ultraviolet rays in the summer sun, set at 5%; *Q* represents the radiation intensity of the ultraviolet lamp (W/m^2^); *t* represents the sufficient time for converting natural radiation to indoor radiation (h).

#### 2.4.3. Coastal Humidity

The coastal humidity condition was determined by considering both summer rainfall and seawater evaporation effects in Shanghai. Summer is the season with the most concentrated rainfall in Shanghai, and rainfall and humid air can promote moisture-related aging of asphalt pavements. In coastal regions, seawater evaporation also leads to a salt-containing humid environment, which is different from ordinary freshwater humidity. Therefore, simulated seawater was used for spraying in this study to reproduce the saline humidity condition of coastal pavement service environments.

The spraying amount was calculated based on the average summer rainfall data of Shanghai from 2001 to 2020. And the simulated spraying volume of the test was calculated to be 182.9 mL. The artificial seawater solution was prepared according to the specification ASTMD 1141-98 [32]. To evaluate the aging and regeneration behavior of SBS-modified asphalt under relatively unfavorable coastal saline conditions, a severe salt concentration condition was selected in this study. It should be noted that the laboratory spraying protocol was designed to simulate the salt-containing humid exposure caused by coastal rainfall and seawater evaporation, rather than to reproduce the complete natural evaporation process.

## 3. Performance of SBS-Modified Asphalt After Aging and Regeneration

### 3.1. High- and Low-Temperature Performance

For aged SBS-modified asphalt, 3%, 6%, and 9% of industrial animal oil and waste engine oil were added, respectively. Under the same preparation and testing conditions, the softening point and ductility of each group of regeneration asphalt samples were measured, and the differences in softening point and ductility between the 0–3%, 3–6%, and 6–9% content stages were calculated as the changes in softening point and ductility. These were used to characterize the changes in the recovery degree of the aging asphalt’s high-temperature performance and the recovery degree of its low-temperature ductility after adding regeneration materials.

As shown in Table 4, the softening point and ductility results indicate that temperature–UV–coastal humidity coupled aging caused more severe deterioration of SBS-modified asphalt than thermal-oxidative aging alone. After thermal-oxidative aging, the softening point was 62.4 °C and the ductility was 43.9 cm. After further coupled aging, the softening point increased to 67.4 °C, while the ductility decreased sharply to 17.7 cm. This indicates that, on the basis of thermal-oxidative aging, the additional effects of UV radiation and coastal humidity further intensified asphalt hardening and reduced the low-temperature deformation capacity of SBS-modified asphalt.

After industrial animal oil and waste engine oil were added as regeneration materials, the softening point of the regeneration asphalt gradually decreased with increasing regeneration material content, whereas the ductility increased continuously. For industrial animal oil, the softening point decreased from 67.4 °C to 54.5 °C as the content increased from 0% to 9%, while the ductility increased from 17.7 cm to 61.9 cm. For waste engine oil, the softening point decreased from 67.4 °C to 57.6 °C, and the ductility increased from 17.7 cm to 48.9 cm. This indicates that both regeneration materials can effectively restore the low-temperature deformation capacity of aged asphalt, but this improvement is accompanied by a certain reduction in high-temperature stability. The decrease in softening point quantitatively reflects the reduction in high-temperature stability caused by the addition of oil-based rejuvenators. At 6% content, the softening point decreased by 9.1 °C for industrial animal oil and 7.5 °C for waste engine oil compared with the non-regenerated aged asphalt. Nevertheless, the softening points of the two regenerated asphalts were 58.3 °C and 59.9 °C, respectively, both of which were higher than the minimum requirement of 55 °C for the Technical Specifications for Construction of Highway Asphalt Pavements (JTG F40-2004) [33].

The different variation trends are mainly related to the component characteristics of the two regenerations. The incorporation of oil-based regenerations replenishes the light components lost during coupled aging, adjusts the colloidal balance of aged asphalt, and improves the mobility of asphalt molecular segments, thereby significantly enhancing ductility. However, the increase in light components also weakens the cohesion of the asphalt colloidal system and reduces the resistance to high-temperature deformation, leading to a continuous decrease in softening point.

Compared with waste engine oil, industrial animal oil shows a stronger effect on ductility recovery but causes a greater reduction in softening point. This may be because industrial animal oil contains more low-molecular-weight fatty acid glycerides and hydrocarbon components, which can more easily penetrate and relax the aged asphalt structure, thus improving low-temperature flexibility more effectively. In contrast, waste engine oil contains more long-chain hydrocarbons, polycyclic aromatic hydrocarbons, and oxidation by-products, with relatively higher viscosity and molecular weight. Therefore, it has a weaker softening effect on the asphalt network and better retention of high-temperature stability, but its ability to improve ductility is relatively limited.

The softening point and ductility of asphalt varies with different oil contents. To further analyze the regeneration effect, the changes in softening point and ductility under different contents were calculated, as shown in Table 5.

As shown in Table 5, the incremental changes in softening point and ductility exhibit different sensitivity characteristics with increasing rejuvenator content. For the softening point, the greatest decrease occurs in the 3–6% content range for both industrial animal oil and waste engine oil, with reductions of 5.5 °C and 4.2 °C, respectively. In contrast, the reductions in the 6–9% range decrease to 3.8 °C and 2.3 °C, indicating that the softening effect of the regenerations gradually weakens when the content exceeds 6%. This suggests that the supplementation of light components tends to approach a saturated state, and further addition of regenerations produces a limited additional softening effect while continuing to weaken the high-temperature stability of asphalt.

For ductility, the largest improvement appears in the initial 0–3% content range. The ductility increases by 23.5 cm with industrial animal oil and by 13.1 cm with waste engine oil, after which the incremental improvement gradually decreases. When the content increases from 6% to 9%, the ductility increments are only 6.5 cm and 6.7 cm, respectively. This indicates that the low-temperature flexibility of aged SBS-modified asphalt is highly sensitive to the initial addition of oil-based regenerations. At low contents, the regeneration processes can effectively replenish the light components lost during coupled aging and improve the mobility of asphalt molecular segments. However, as the rejuvenator content further increases, the colloidal structure gradually reaches a new equilibrium, and the marginal improvement in ductility becomes limited.

Comparing the two regeneration conditions, industrial animal oil produces larger changes in both softening point and ductility, especially in the 0–6% range, indicating a stronger softening and low-temperature recovery effect. Waste engine oil shows a relatively smaller reduction in softening point and a lower ductility improvement, suggesting that it has a milder regeneration effect and better retention of high-temperature stability. Therefore, the regeneration effect of oil-based regenerations should be evaluated by balancing the improvement in ductility and the reduction in softening point. Excessive addition is not necessarily beneficial, because the additional improvement in ductility becomes limited while the high-temperature stability continues to decline.

### 3.2. Viscosity Result

Aging SBS-modified asphalt was respectively mixed with 3%, 6%, and 9% of industrial animal oil and waste engine oil. Under the same preparation and testing conditions, the viscosity of each group of regeneration asphalt samples at 135 °C was tested. The viscosity difference between 0–3%, 3–6%, and 6–9% of each contents stage was calculated as the viscosity change value. This characterizes the recovery degree of the aging asphalt rheological properties after the addition of regeneration materials. The test results are shown in Table 6.

**Figure 9:**
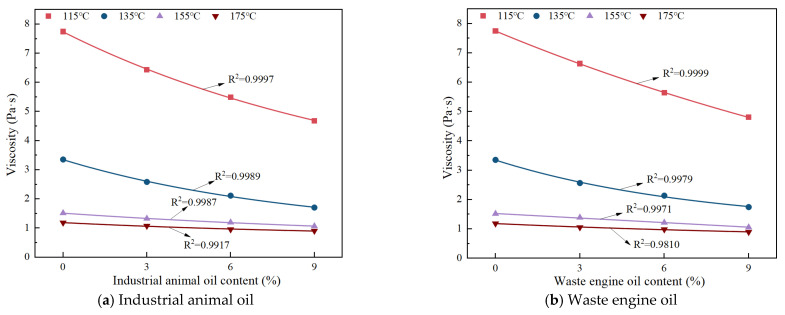
Fitting curves for viscosity at various temperatures.

As shown in Figure 9, the viscosity of regenerated SBS-modified asphalt decreases with increasing rejuvenator content at all test temperatures, indicating that both industrial animal oil and waste engine oil can improve the flowability of coupled-aged asphalt. At the same rejuvenator content, the viscosity decreases markedly with increasing temperature, which confirms that the regenerated asphalt still maintains typical temperature-sensitive rheological behavior. The high fitting coefficients indicate that the fitted curves can describe the viscosity variation well. Overall, the two rejuvenators show similar viscosity-reduction trends.

As shown in Table 6, the viscosity results indicate that temperature–UV–coastal humidity coupled aging caused more severe hardening of SBS-modified asphalt than thermal-oxidative aging alone. After thermal-oxidative aging, the viscosities at 115 °C, 135 °C, 155 °C, and 175 °C were 6.24 Pa·s, 2.51 Pa·s, 1.02 Pa·s, and 0.72 Pa·s, respectively. After further coupled aging, the corresponding viscosities increased to 7.74 Pa·s, 3.35 Pa·s, 1.51 Pa·s, and 1.18 Pa·s. This indicates that, on the basis of thermal-oxidative aging, the additional effects of UV radiation and coastal humidity further increased the viscosity of SBS-modified asphalt and reduced its flowability.

After the SBS-modified asphalt was subjected to temperature-UV-humidity coupled aging, when 3%, 6%, and 9% of industrial animal oil and waste engine oil were respectively added, the rotational viscosities of the asphalt all showed a significant downward trend. This result indicates that both of the regeneration materials can effectively improve the fluidity and workability of the aged asphalt. From the perspective of the regeneration mechanism, industrial animal oil and waste engine oil contain abundant light components, which can replenish the oil lost due to volatilization and oxidation reactions during the aging process and restore the colloid structure balance among oil, resin, and asphaltene in the asphalt system, thereby improving the macroscopic flow performance and showing good regeneration effects.

As the test temperature increased from 115 °C to 175 °C, the viscosity of all asphalt samples decreased significantly, demonstrating the typical temperature sensitivity of asphalt binders. Although coupled aging increased the viscosity compared with thermal-oxidative aging alone, the viscosity–temperature variation trend remained consistent. After the addition of industrial animal oil and waste engine oil, the viscosity–temperature curves shifted downward, while the basic temperature-dependent rheological behavior was still maintained. This suggests that the two regeneration materials mainly improve the flow properties of aged asphalt by supplementing light components and regulating the colloidal balance, without fundamentally changing the thermal rheological characteristics of SBS-modified asphalt.

To further analyze the regeneration effect, the change in viscosity at different concentrations was calculated, as shown in Table 7.

As shown in Table 7, as the proportion of regeneration materials increases, the overall change in viscosity of regeneration asphalt shows a downward trend. When the proportion is 3%, regardless of whether industrial animal oil or waste engine oil is used, the viscosity reduction is the most significant. This indicates that at this stage, the regeneration materials are functioning most effectively, effectively adjusting the component ratio. As the proportion increases to 6% and 9%, the rate of viscosity reduction gradually slows down. At this point, the supplementation of light components has approached a saturated state, indicating that the decrease in viscosity gradually slows down with the increase in proportion. At the same time, if too much regeneration material is added, excessive regeneration material may cause changes in the structural system, thereby affecting the stability of the regeneration asphalt. Therefore, the proportion should be controlled within a reasonable range.

From the perspective of the type of regeneration materials, the viscosity changes for industrial animal oil regeneration asphalt are generally lower than those for waste engine oil regeneration asphalt. This indicates that industrial animal oil has a stronger ability to regulate viscosity and a more significant regeneration effect. This is because industrial animal oil mainly comes from restaurant waste oil and by-products of animal fat processing, which is rich in polar organic substances such as fatty acid esters and glycerol esters. The polar groups in the molecular structure can interact with the highly polar oxygen-containing functional groups in aged asphalt, promoting the penetration, dispersion and uniform distribution of the regenerant, thereby more effectively reducing the viscosity of the system.

In contrast, although used engine oil contains a relatively large amount of light hydrocarbon components, which can to some extent replenish the missing light components in aged asphalt, its molecular polarity is relatively weak, and its interaction with the polar oxidative products in aged asphalt is limited. The above research results indicate that both industrial animal oil and used engine oil can significantly improve the rheological properties of SBS-modified asphalt after temperature–ultraviolet–humidity coupled aging. However, industrial animal oil has a more advantageous polarity feature in terms of viscosity recovery and structure regulation, while the effect of used engine oil is second.

### 3.3. Determination of the Optimal Content of Regeneration Materials

This study conducted a regression analysis on the ductility and viscosity (at 135 °C) of asphalt under two types of regeneration materials. With the recovery of the conventional physical properties of regeneration asphalt to the level of un-aged SBS-modified asphalt as the reference, the theoretical optimal blending ratios of each regeneration material were calculated based on the obtained regression equation. The results are shown in Table 8.

As shown in Table 8, the theoretical optimal contents calculated from the regression equations show clear differences between industrial animal oil and waste engine oil. For industrial animal oil, the optimal contents corresponding to ductility and viscosity are 7.03% and 4.89%, respectively, indicating that industrial animal oil can simultaneously contribute to the recovery of low-temperature ductility and viscosity regulation of coupled-aged SBS-modified asphalt. The difference between these two optimal values also suggests that the recovery of different performance indicators does not occur at exactly the same rejuvenator content. Therefore, the content should be determined by considering the coordinated recovery of multiple properties rather than a single index.

For waste engine oil, a relatively clear optimal content of 5.65% is obtained based on the viscosity index, while no effective optimal content is calculated for ductility. This indicates that waste engine oil can effectively reduce the viscosity and improve the flowability of aged asphalt, but its ability to restore low-temperature ductility is limited. This result is consistent with the previous analysis, in which waste engine oil showed a weaker ductility improvement than industrial animal oil under the same content. The difference may be attributed to the relatively higher molecular weight and viscosity of waste engine oil, which makes it less effective in relaxing the aged asphalt colloidal structure and restoring molecular mobility.

In addition, the softening point results indicate that increasing the content of regeneration materials continuously weakens the high-temperature stability of asphalt. Therefore, although the regression analysis gives different theoretical optimal contents for different indicators, excessive addition is not suitable from the perspective of high-temperature performance retention. Combining the regression results of ductility and viscosity with the variation trends of the softening point, the practical recommended content of oil-based regenerations would be around 6%. At this content level, the regenerated asphalt can achieve a relatively balanced improvement in ductility and viscosity while avoiding excessive softening. It should be emphasized that the 6% content was determined as a practical recommended content based on the tested content gradient and the coordinated consideration of ductility, viscosity, and softening point, rather than as a universal optimum obtained from a weighted multi-objective optimization model. The regression results show that the optimal contents for different indicators are not identical, and the two rejuvenators exhibit different recovery characteristics. Therefore, the recommended content of approximately 6% is applicable to the coupled-aged SBS-modified asphalt and the content range investigated in this study.

## 4. Regeneration Mechanisms of Coupled-Aged SBS-Modified Asphalt

From the above analysis, it can be concluded that the optimal combined blending ratio of industrial animal oil and waste engine oil for regeneration is set at 6%. Therefore, in the research on the regeneration mechanism, an analysis was conducted using a mixture of industrial animal oil and waste engine oil with a 6% ratio.

### 4.1. Functional Group Law

**Figure 10:**
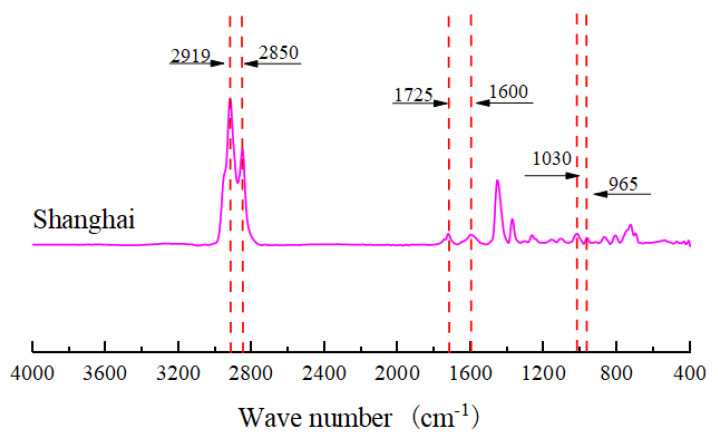
Infrared spectrum of industrial animal oil regeneration asphalt.

**Figure 11:**
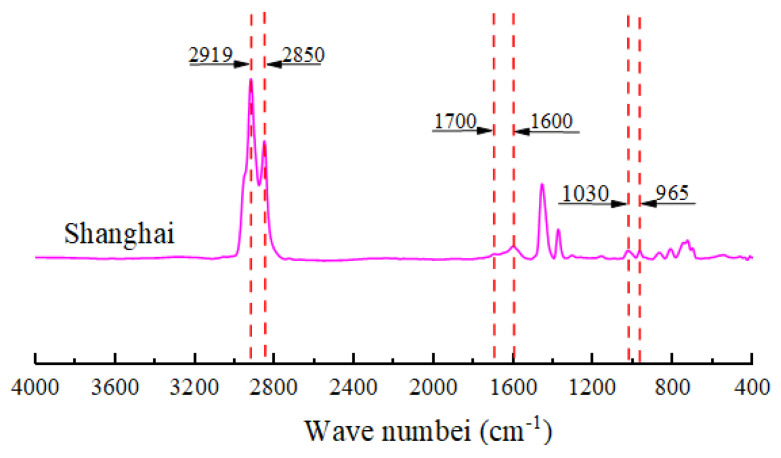
Infrared spectrum of waste engine oil regeneration asphalt.

The changes in *CI*, *SI*, and *BI* at a 6% concentration were analyzed through infrared spectroscopy. The infrared spectroscopy results of the regeneration of industrial animal oil and waste engine oil are shown in Figure 10 and Figure 11.

The OMNIC 9.2 and PeakFit V4.12 software packages were used to fit and obtain the area of the chemical functional group absorption peaks of the SBS-modified asphalt after regeneration. Then, Equations (5)–(7) were used to calculate the corresponding *CI*, *SI*, and *BI* of the asphalt before and after aging. The calculation results of industrial animal oil and waste engine oil regeneration are shown in Figure 3.

(5) $$CI=\frac{A_{1700}}{A}$$

(6) $$SI=\frac{A_{1030}}{A}$$

(7) $$BI=\frac{A_{965}}{A}$$

where A_1700_ represents the peak area at 1700 cm^−1^ and indicates the content of C=O; A_1030_ represents the peak area at 1030 cm^−1^ and indicates the content of S=O; A_966_ represents the peak area at 966 cm^−1^ and indicates the content of SBS; A is the sum of the peak areas within the range of 600–2000 cm^−1^.

**Figure 12:**
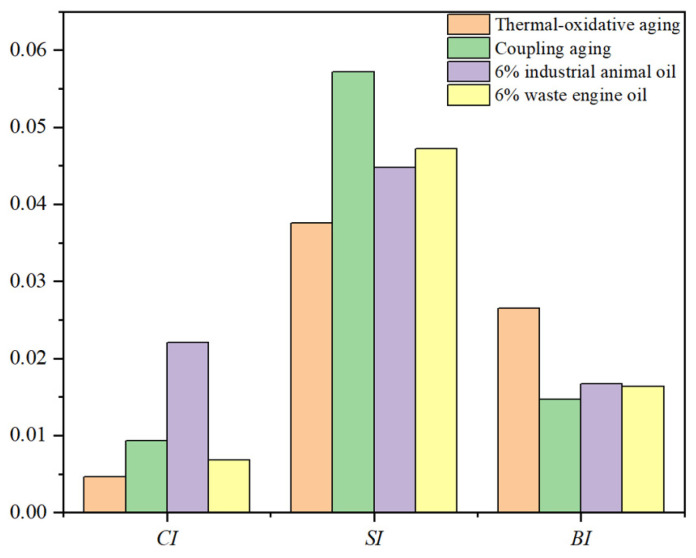
*CI*, *SI*, and *BI* of regeneration asphalt.

As shown in Figure 12, the functional group indices indicate that temperature–UV–coastal humidity coupled aging caused more severe chemical deterioration of SBS-modified asphalt than thermal-oxidative aging alone. Compared with the thermal-oxidative aged asphalt, the coupled-aged asphalt shows higher *CI* and *SI* values, indicating that the additional effects of UV radiation and coastal humidity further promote the formation and accumulation of carbonyl and sulfoxide groups. Meanwhile, the *BI* value decreases after coupled aging, suggesting that the SBS-related butadiene structure is further affected under the combined environmental exposure. This indicates that thermal-oxidative aging provides the basic oxidation process, while the subsequent temperature–UV–coastal humidity coupled aging further intensifies asphalt oxidation and SBS structural degradation.

After adding 6% industrial animal oil and 6% waste engine oil as regeneration materials, the functional group indices of coupled-aged SBS-modified asphalt change to different degrees. Compared with the non-regenerated coupled-aged asphalt, the *SI* values of both regeneration asphalts decrease significantly, indicating that the two oil-based regeneration materials can effectively reduce the apparent contribution of S=O-related polar functional groups. This is mainly because the light components introduced by the regeneration materials dilute and redistribute the polar fractions accumulated during aging, thereby improving the colloidal dispersion state of the aged asphalt system. Among the three indices, *SI* exhibits the most consistent response to both regeneration materials, suggesting that it is more sensitive in characterizing the regeneration effect.

The *CI* values show different variation trends for the two regenerations. After adding industrial animal oil, *CI* increases markedly, whereas waste engine oil slightly decreases the *CI* value. The increase in *CI* for industrial animal oil does not necessarily indicate further oxidation of asphalt. Instead, it may be related to the carbonyl-containing compounds in industrial animal oil, such as fatty acid glycerides or ester groups, whose characteristic absorption near 1700 cm^−1^ overlaps with that of the asphalt matrix. Therefore, the *CI* value in the industrial animal oil rejuvenated system should be interpreted as an apparent carbonyl-related absorption intensity rather than a direct indicator of oxidation degree. In contrast, waste engine oil mainly contains hydrocarbon-based light fractions, which can dilute the relative concentration of oxidation products in aged asphalt, leading to a slight decrease in *CI*.

For the *BI* index, both industrial animal oil and waste engine oil result in a slight increase compared with the non-regenerated asphalt. This indicates that the two regenerations can improve the dispersion state or apparent continuity of SBS-related structures to a certain extent. The introduced light fractions may exert swelling and lubricating effects on the residual SBS phase, promoting partial relaxation and redistribution of SBS components within the asphalt matrix. However, the limited increase in BI also suggests that the regeneration process is mainly governed by physical swelling, component redistribution, and compatibility improvement, rather than substantial chemical reconstruction of SBS molecular chains.

No new characteristic absorption peaks were observed after regeneration, and the changes in *CI*, *SI*, and *BI* mainly reflected the redistribution or dilution of oxidation-related polar groups and the apparent response of SBS-related structures. Therefore, within the resolution of FTIR analysis, no direct evidence of new chemical bonding or substantial chemical reconstruction of SBS chains was obtained. Previous studies have indicated that oil-based or component-regulating rejuvenators usually restore aged SBS-modified asphalt mainly through light component supplementation, colloidal structure regulation, and swelling of residual SBS phases, whereas the chemical reconnection of degraded SBS chains generally requires reactive functional groups, such as epoxy or isocyanate groups [29,34,35]. Overall, industrial animal oil and waste engine oil both regulate the chemical characteristics of coupled-aged SBS-modified asphalt through light component replenishment and colloidal structure adjustment, but their effects on specific functional group indices are different. Industrial animal oil shows a stronger influence on *CI* due to its external carbonyl-containing components, while waste engine oil produces a milder *CI* response and mainly acts through hydrocarbon-based dilution and dispersion. The common decrease in *SI* and slight increase in *BI* confirm that the regeneration mechanism of both oils is dominated by polar component redistribution, colloidal system rebalancing, and physical improvement of SBS phase dispersion.

### 4.2. Microscopic Morphological Characteristics

**Figure 13:**
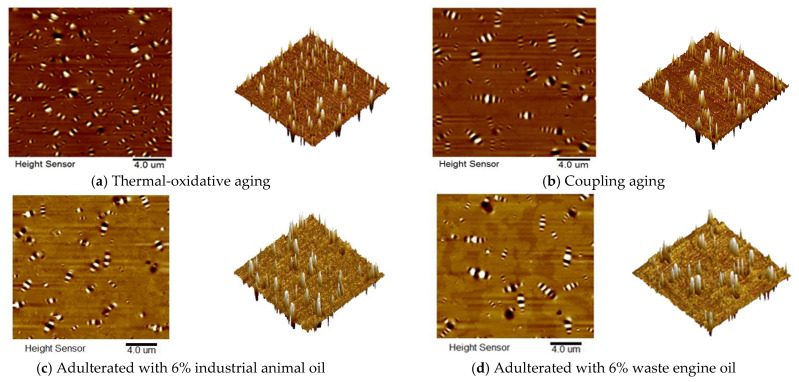
Microscopic morphology of industrial animal oil and waste engine oil regeneration asphalt.

In this study, AFM was used to observe the microscopic structure of regeneration asphalt at different concentrations, and to analyze its microscopic morphology and surface roughness. The data obtained by AFM were processed and analyzed using NanoScope Analysis 1.5 and Image-Pro Plus 7 software, resulting in the surface morphology images of industrial animal oil and waste engine oil regeneration asphalt at a 6% concentration, as shown in Figure 13. The detailed parameters of the “bee-like structure” based on image analysis are presented in Table 9.

As shown in Figure 13, the AFM morphology results indicate that temperature–UV–coastal humidity coupled aging caused more severe microstructural deterioration of SBS-modified asphalt than thermal-oxidative aging alone. After thermal-oxidative aging, the asphalt surface already exhibits a certain degree of roughness and dispersed protruding “bee-like structures”, indicating the aggregation of polar components and the development of micro-phase separation in the aged asphalt system [36]. After further coupled aging, the surface becomes rougher, and the protruding “bee-like structures” appear more developed and densely distributed, suggesting that, on the basis of thermal-oxidative aging, the additional effects of UV radiation and coastal humidity further intensify the aggregation of polar components and the heterogeneity of the asphalt micro-phase structure.

After the incorporation of 6% industrial animal oil and 6% waste engine oil, the AFM morphology of the coupled-aged SBS-modified asphalt changes significantly. The surface morphology becomes more uniform, and the number and size of the “bee-like structures” are markedly reduced, indicating that both oil-based regeneration materials can effectively regulate the micro-phase structure of aged asphalt. This suggests that the two regeneration materials can disperse the aggregated domains formed during coupled aging and promote the transition of the asphalt system toward a more homogeneous microstructural state.

The statistical results in Table 9 further confirm the AFM morphological observations. After thermal-oxidative aging, the asphalt exhibits 146 “bee-like structures”, with a mean area of 74.83 μm^2^ and an area ratio of 4.75%. After further temperature–UV–coastal humidity coupled aging, the number of “bee-like structures” decreases to 111, whereas the mean area increases significantly to 114.00 μm^2^, indicating that, on the basis of thermal-oxidative aging, the additional effects of UV radiation and coastal humidity promote the aggregation and coarsening of the micro-phase domains. At the same time, the area ratio decreases slightly to 4.38%, suggesting that the distribution of the “bee-like structures” becomes less dense but more developed in size.

After the addition of 6% industrial animal oil and 6% waste engine oil, the “bee-like structure” parameters change significantly. For industrial animal oil, the number of “bee-like structures” decreases from 111 to 65, the mean area decreases from 114.00 μm^2^ to 64.17 μm^2^, and the area ratio decreases from 4.38% to 3.38%. For waste engine oil, the number further decreases to 59, and the mean area decreases to 57.76 μm^2^, while the area ratio slightly increases to 4.69%. These results indicate that both regeneration materials can effectively suppress the development of aggregated domains formed during coupled aging and promote the refinement of the micro-phase structure. Compared with industrial animal oil, waste engine oil shows a slightly stronger effect in reducing the number and size of “bee-like structures”, whereas industrial animal oil exhibits a more pronounced reduction in area ratio.

However, the area ratio exhibits different variation characteristics for the two regenerations. After adding industrial animal oil, the area ratio decreases from 4.38% to 3.38%, indicating that industrial animal oil effectively weakens the overall aggregation degree of “bee-like structures”. In contrast, the area ratio of waste engine oil slightly increases to 4.69%, although its number and mean area decrease. This phenomenon may be attributed to the fragmentation and redistribution of large aggregated domains into smaller dispersed structures, some of which are still retained in the image segmentation results. Therefore, the slight increase in the area ratio does not contradict the reduction in number and mean area but reflects a refined and redistributed micro-phase morphology [37].

The above changes are mainly related to the light components introduced by the regenerations. Industrial animal oil and waste engine oil can replenish the light fractions lost during coupled aging, dilute and disperse asphaltene-rich or wax-related aggregated structures and improve the colloidal balance of aged asphalt. As a result, the originally rough and heterogeneous microstructure gradually evolves toward a more dispersed and homogeneous state. Compared with industrial animal oil, waste engine oil may contain more aromatic and soluble light components, giving it a stronger swelling and dispersion effect on aggregated domains. Overall, the AFM results indicate that both oil-based regenerations improve the micro-morphology of coupled-aged SBS-modified asphalt mainly through light component replenishment, colloidal structure regulation, and physical dispersion, rather than chemical reconstruction of the asphalt or SBS molecular structure.

Furthermore, using the “Roughness” module in the Nanoscope Analysis 1.5 software, the asphalt mixed with industrial animal oil and waste engine oil was calculated to obtain the root mean square roughness (Rq) and arithmetic mean roughness (Ra), as shown in Table 10.

It should be noted that the Rq and Ra obtained from AFM analysis are micro-morphological characterization indices rather than standard quality-control indicators for asphalt binders. Current asphalt pavement specifications do not provide unified tolerance limits for AFM roughness parameters or bee-like structure characteristics. Nevertheless, previous studies have widely used AFM-derived roughness indices and bee-like structure parameters to characterize the aging and regeneration behavior of asphalt binders [38,39,40]. Following these studies, all samples in this work were tested and processed under identical AFM conditions, including scanning mode, scan size, probe type, and image-processing procedure.

As shown in Table 10, the surface roughness parameters indicate that temperature–UV–coastal humidity coupled aging caused more severe micro-topographical deterioration of SBS-modified asphalt than thermal-oxidative aging alone. After thermal-oxidative aging, the Rq and Ra values were 5.20 and 2.68, respectively. After further coupled aging, these values increased to 6.81 and 3.21, indicating that, on the basis of thermal-oxidative aging, the additional effects of UV radiation and coastal humidity further increased the surface roughness and intensified the heterogeneity of the asphalt microstructure.

Under the 6% content condition, both industrial animal oil and waste engine oil effectively reduce the surface roughness parameters (Rq and Ra) of aged SBS-modified asphalt, indicating that the incorporation of regenerations significantly improves the micro-scale surface topography, transforming it from an originally rough and heterogeneous state toward a smoother and more homogeneous morphology.

Specifically, the Rq and Ra values of the industrial animal oil system are 5.96 and 2.97, respectively, whereas those of the waste engine oil system decrease to 4.89 and 2.36. This demonstrates that both regenerations exhibit a clear smoothing effect on the asphalt surface. However, waste engine oil shows a more pronounced reduction in both Rq and Ra, indicating a stronger capability to reconstruct the micro-surface structure of aged asphalt.

This phenomenon can be attributed to the shared mechanism of both regenerations, namely, the replenishment of light fractions (saturates and aromatics), which improves the colloidal balance of aged asphalt depleted by oxidative aging. As a result, surface protrusions formed by the aggregation of highly polar asphaltenes are gradually weakened and filled, leading to a reduction in surface undulations and an overall decrease in roughness. Meanwhile, this trend is consistent with the variation in “bee-like structure” parameters discussed previously, where the reduction in bee-like structures corresponds well with the decrease in surface roughness, jointly reflecting the transition of the microstructure from an aggregated state to a more dispersed state during the regeneration process.

Overall, the roughness analysis results indicate that both regenerations significantly improve the surface morphology of aged SBS-modified asphalt; however, waste engine oil exhibits superior performance in terms of surface smoothing. This observation is in good agreement with the AFM morphology analysis and bee-like structure statistical results. These AFM results further support the physical-dominated regeneration mechanism. The reduction in the number, mean area, and roughness of bee-like structures indicates that the oil-based rejuvenators mainly disperse asphaltene-rich aggregated domains and improve the colloidal morphology of aged asphalt, rather than directly repairing the molecular chains of degraded SBS.

### 4.3. SBS Structural Characteristics

**Figure 14:**
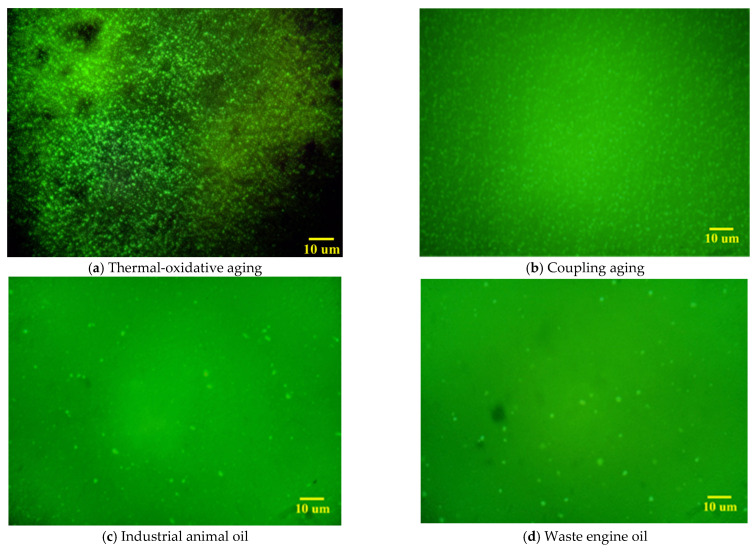
Image of regeneration asphalt with industrial animal oil and waste engine oil.

This study utilized FM to conduct an observation and analysis of regeneration asphalt. By comparing and analyzing the fluorescence characteristics, SBS distribution, and structural changes, the recovery mechanism of regeneration materials for aged asphalt is revealed. As shown in Figure 14.

As shown in Figure 14, the fluorescence images indicate that temperature–UV–coastal humidity coupled aging caused more severe degradation of the SBS phase structure than thermal-oxidative aging alone. After thermal-oxidative aging, distinct SBS-related fluorescent domains can still be observed, whereas after coupled aging, the fluorescence image becomes more uniform and diffuse, and the SBS-related phase is significantly weakened. This indicates that, on the basis of thermal-oxidative aging, the additional effects of UV radiation and coastal humidity further accelerate the degradation of SBS components and the destruction of the polymer network structure [41,42]. After adding industrial animal oil and waste engine oil, the dispersed SBS-related fluorescence phase becomes more visible again, and its local continuity is partially restored, indicating that both regeneration materials can improve the phase distribution state of residual SBS components. Compared with industrial animal oil, waste engine oil shows a slightly stronger effect on the recovery of SBS phase dispersion. However, the regenerated SBS phase still remains less continuous than that of the thermal-oxidative aged asphalt, suggesting that the recovery is mainly dominated by physical redistribution and swelling of residual SBS components rather than complete molecular reconstruction.

With the addition of industrial animal oil or waste engine oil, the morphology of the SBS-related phase in the fluorescence images is noticeably improved, as evidenced by a partial recovery of dispersed phase structures and enhanced local continuity. This suggests that the incorporation of oil-based rejuvenators facilitates the swelling and re-dispersion of residual SBS components to a certain extent. However, compared with the original SBS polymer network, the regenerated SBS structure still exhibits reduced size, non-uniform distribution, and insufficient connectivity. Therefore, the observed fluorescence recovery should be interpreted as an improvement in the physical state and phase distribution of residual SBS components, rather than full reconstruction of scissored SBS molecular chains. This interpretation is consistent with previous studies showing that conventional oil-based rejuvenators mainly improve the compatibility and dispersion of residual SBS phases, while true SBS chain reconnection generally requires reactive regeneration materials with specific functional groups [43].

Further comparison reveals clear differences in the recovery degree of SBS structures between industrial animal oil and waste engine oil. The waste engine oil system exhibits a more continuous and uniformly distributed fluorescence phase, indicating a more effective swelling and dispersion of the SBS phase. This may be attributed to the higher content of aromatic fractions and soluble light components in waste engine oil, which can more effectively penetrate the interface between the SBS phase and the asphalt matrix, thereby enhancing compatibility and promoting partial restoration of the SBS network structure.

Overall, the fluorescence microscopy results demonstrate that both industrial animal oil and waste engine oil can improve the distribution state of the SBS phase in aged SBS-modified asphalt to a certain extent. However, this process is essentially governed by physical swelling and phase restructuring rather than chemical reconstruction of polymer chains [44]. Among them, waste engine oil exhibits a more pronounced effect on restoring the SBS network structure, which is in good agreement with the AFM morphology, bee-like structure analysis, and surface roughness results discussed previously.

## 5. Conclusions

In this study, a temperature-UV-coastal humidity coupled aging test system was established. The system studied the recovery laws and microscopic action mechanisms of the performance of coupled-aged SBS-modified asphalt by industrial animal oil and waste engine oil. The following conclusions were obtained:

- The addition of the industrial animal oil and waste engine oil can significantly improve the low-temperature ductility and rheological properties of the coupled-aged SBS-modified asphalt, but it will also reduce its high-temperature softening point performance.
- As the concentration of the industrial animal oil and waste engine oil increased from 3% to 9%, the recovery of asphalt properties shows a pattern of significant improvement initially and then reaching a saturation point. A content of approximately 6% was recommended as a practical content for both industrial animal oil and waste engine oil to balance ductility recovery, viscosity reduction, and high-temperature stability under the tested coupled-aging condition.
- Industrial animal oil and waste engine oil exhibited different performance recovery characteristics. Industrial animal oil showed a stronger effect on ductility recovery, indicating a better ability to improve low-temperature flexibility. In contrast, waste engine oil showed a more moderate softening effect and a more obvious role in viscosity regulation, suggesting better retention of high-temperature stability and stronger flowability adjustment.
- FTIR, AFM, and fluorescence microscopy results indicate that no evidence of substantial chemical reconstruction of SBS molecular chains was observed within the applied characterization methods. The regeneration process is mainly associated with light component replenishment, redistribution of polar components, dispersion of asphaltene-rich aggregated domains, colloidal structure regulation, and improvement of residual SBS phase dispersion.
- Industrial animal oil and waste engine oil showed different microscopic regulation effects. Industrial animal oil had a stronger influence on carbonyl-related functional groups, whereas waste engine oil was more effective in reducing bee-like structures and improving SBS phase dispersion, indicating different roles in microstructure regulation.
- From an environmental perspective, both industrial animal oil and waste engine oil show potential as waste-derived asphalt rejuvenators, which can promote resource recycling and reduce the consumption of virgin petroleum-based materials. However, the two products should not be treated as completely equivalent in industrial applications. Industrial animal oil is more favorable for improving low-temperature ductility and has advantages in waste lipid utilization, whereas waste engine oil is more effective in viscosity regulation and microstructure adjustment but requires stricter pretreatment and environmental safety control due to its possible contaminants and oxidation by-products. Therefore, their industrial application should be based on separate performance evaluation, source stability, pretreatment quality, and environmental risk assessment.

In future studies, more field monitoring data (UV intensity and spectrum, salt spray/humidity concentration, temperature cycles, exposure duration) will be collected from coastal areas to establish a more reliable connection between the laboratory and the field. The dynamic shear rheological indices (e.g., changes in rutting factor, PG grade, and creep compliance) and the long-term re-aging behavior of the modified binders will be analyzed in future studies to more comprehensively evaluate the anti-rutting ability and long-term engineering application.

## Figures and Tables

**Table 1:** Performance indicators of SBS-modified asphalt.

Index	Unit	Standard Requirement	Test Result
Needle penetration (25 °C, 100 g, 5 s)	mm	40~60	57
Softening point	°C	>75	82.5
Ductility at 5 °C	cm	>20	61.4
Elastic recovery (25 °C)	%	≥75	92
Kinematic viscosity (135 °C)	Pa·S	<3	2.01
Flash point (Open)	°C	>230	329
Solubility (trichloroethylene)	%	>99	99.74
Density (15 °C)	g/cm^3^	/	1.033

**Table 2:** Performance indicators of industrial animal oil.

Test Items	Unit	Index
Appearance	-	Pale yellow
Kinematic viscosity (40 °C)	mm^2^/s	30–50
Flash point	°C	260
Density (20 °C)	g/mL	0.95

**Table 3:** Main technical properties of G-CR.

Test Items	Unit	Index
Appearance	-	Pale yellow
Kinematic viscosity (40 °C)	mm^2^/s	42.1
Kinematic viscosity (100 °C)	mm^2^/s	8.0
Flash point	°C	222
Density (15 °C)	kg/m^3^	834.7

**Table 4:** Test results of softening point and ductility of regeneration asphalt.

Test	Aging/Regeneration Condition	Regenerated Content (%)	Test Result
Softening point	Thermal-oxidative aging	/	62.4
	Industrial animal oil	0	67.4
		3	63.8
		6	58.3
		9	54.5
	Waste engine oil	0	67.4
		3	64.1
		6	59.9
		9	57.6
Ductility	Thermal-oxidative aging	/	43.9
	Industrial animal oil	0	17.7
		3	41.2
		6	55.4
		9	61.9
	Waste engine oil	0	17.7
		3	30.8
		6	42.2
		9	48.9

**Table 5:** The results of the change in softening point and ductility of regeneration asphalt.

Test	Regeneration Material	Content (%)	Change
Softening point	Industrial animal oil	0–3	3.6
		3–6	5.5
		6–9	3.8
	Waste engine oil	0–3	3.3
		3–6	4.2
		6–9	2.3
Ductility	Industrial animal oil	0–3	23.5
		3–6	14.2
		6–9	6.5
	Waste engine oil	0–3	13.1
		3–6	10.7
		6–9	6.7

**Table 6:** Test results of viscosity of industrial animal oil and waste engine oil regeneration asphalt.

Temperature (°C)	Thermal-Oxidative Aging	Industrial Animal Oil				Waste Engine Oil			
		0%	3%	6%	9%	0%	3%	6%	9%
115	6.24	7.74	6.43	5.49	4.68	7.74	6.63	5.64	4.80
135	2.51	3.35	2.58	2.11	1.7	3.35	2.56	2.13	1.74
155	1.02	1.51	1.32	1.19	1.06	1.51	1.38	1.21	1.05
175	0.72	1.18	1.07	0.95	0.9	1.18	1.05	0.98	0.89

**Table 7:** Result of viscosity change of regeneration asphalt.

Regeneration Material	Regeneration Evaluation Index	Content (%)	Change
Industrial animal oil	Change in viscosity (Pa·s)	0–3	0.77
		3–9	0.47
		6–9	0.41
Waste engine oil	Change in viscosity (Pa·s)	0–3	0.79
		3–9	0.43
		6–9	0.39

**Table 8:** Optimal content for regular performance indicators of regeneration materials.

Regeneration Materials	Index	Regression Equation
Industrial animal oil	Ductility	y = 17.78 + 9.14x − 0.47x^2^
	Viscosity	y = 3.34 − 0.27x + 0.01x^2^
Waste engine oil	Ductility	y = 16.57 + 5.5x − 0.24x^2^
	Viscosity	y = 3.33 − 0.28x + 0.01x^2^

**Table 9:** Parameters of the “bee-like structure” in regeneration asphalt.

Regeneration	Thermal-Oxidative Aging			Coupling Aging			6% Content		
	Amount	Mean Area (μm^2^)	Area Ratio (%)	Amount	Mean Area (μm^2^)	Area Ratio (%)	Amount	Mean Area (μm^2^)	Area Ratio (%)
Industrial animal oil	146	74.83	4.75	111	114.00	4.38	65	64.17	3.38
Waste engine oil	146	74.83	4.75	111	114.00	4.38	59	57.76	4.69

**Table 10:** Calculation results of Rq and Ra.

Regeneration Material	Thermal-Oxidative Aging		Coupling Aging		6% Content	
	Rq	Ra	Rq	Ra	Rq	Ra
Industrial animal oil	5.20	2.68	6.81	3.21	5.96	2.97
Waste engine oil	5.20	2.68	6.81	3.21	4.89	2.36

## Data Availability

The original contributions presented in this study are included in the article. Further inquiries can be directed to the corresponding author.
